# Ecological and demographic impacts of a recent volcanic eruption on two endemic patagonian rodents

**DOI:** 10.1371/journal.pone.0213311

**Published:** 2019-03-07

**Authors:** Eileen A. Lacey, Risa Takenaka, Katie LaBarbera, Mauro N. Tammone

**Affiliations:** 1 Museum of Vertebrate Zoology and Department of Integrative Biology, University of California, Berkeley, CA, United States of America; 2 Programa de Estudios Aplicados a la Conservación del Parque Nacional Nahuel Huapi, Bariloche, Río Negro, Argentina; Université de Sherbrooke, CANADA

## Abstract

Catastrophic events can significantly impact the demographic processes that shape natural populations of organisms. However, linking the outcomes of such events to specific demographic parameters is often challenging due to a lack of detailed pre-event data. The eruption of the Puyehue-Cordon Caulle volcanic complex on 4 June 2011 had profound consequences for the biota of southwestern Argentina. Our long-term behavioral, ecological, and demographic studies of two species of tuco-tucos (*Ctenomys sociabilis* and *C*. *haigi*) that occur in the region most heavily impacted by ash fall from the eruption provided an unusual opportunity to assess the effects of this event on natural populations of mammals. The post-eruption density of the study population for each species was markedly reduced compared to pre-eruption values, with the relative magnitude of this reduction being greater for the group-living *C*. *sociabilis*. The more extensive data set for this species indicated that ash fall from the eruption altered the food resources available to these animals; differences in pre- and post-eruption stable isotope signatures for fur samples from *C*. *sociabilis* were consistent with observed changes in vegetation. Per capita female reproductive success was also reduced in this species during the first breeding season following the eruption. Based on our detailed demographic records for *C*. *sociabilis*, neither survival of yearling females from 2010 to 2011 nor the percentage of unmarked females in the study population in 2011 differed from pre-eruption values. Instead, the post-eruption decrease in population density for *C*. *sociabilis* appeared to reflect reduced within-population recruitment of juvenile females to the 2011 breeding population. Although the eruption did not result in the local extinction of either study population, the demographic consequences detected are likely to have impacted the effective sizes of these populations, creating important opportunities to link specific demographic parameters to previously reported decreases in genetic variability detected after this significant natural event.

## Introduction

Catastrophic natural events can have profound impacts on multiple aspects of a species’ biology, including demography and associated patterns of genetic diversity [1–3]. Such effects may be dramatic, resulting in extreme reductions in population size, severe genetic bottlenecks and, in some cases, local extinctions [4,5]. Alternatively, these effects may be subtler and slower to emerge, such as changes in population structure or disruptions of dispersal corridors that reduce migration and gene flow and increase the potential for localized genetic drift [5,6]. Each of these outcomes may alter not only current abundance and diversity but also–due to their effects on demography and genetic structure—the evolutionary trajectories of affected species [7,8].

To understand why genetic and other outcomes of catastrophic events vary, it is necessary to identify the specific demographic parameters and processes affected by such occurrences [9,10]. Obtaining the requisite data, however, can be challenging–most natural catastrophes are unpredictable, making it difficult to design research programs to assess the impacts of these phenomena on local biota. In particular, it is rare to have detailed pre- and post-event data that can be used to link abrupt environmental changes to specific demographic parameters that may affect other aspects of an organism’s biology [11]. Robust pre-event data may be particularly important when assessing the consequences of less extreme environmental changes, since the consequences of these changes may be more difficult to detect from post-event data alone.

Studies of ctenomyid rodents from the Limay Valley of northwestern Patagonia provide a rare opportunity to assess the consequences of a potentially catastrophic event on the demographic structures of natural populations of vertebrates. Commonly known as tuco-tucos, these subterranean animals spend nearly all of their lives in underground burrow systems [12,13]. Two species of ctenomyids occur in the Limay Valley and surrounding hills. The Colonial tuco-tuco (*Ctenomys sociabilis*), which occurs west of the Río Limay, is a habitat specialist that occupies mesic patches in the otherwise arid steppe grassland that predominates in this region [14,15]. In contrast, the Patagonian tuco-tuco (*C*. *haigi*) is a habitat generalist that is widely distributed along the eastern side of the Río Limay and areas to the north and south [14,16]. Although populations of these species occur in similar habitats located in close proximity to one another, these taxa differ markedly with respect to social structure; while the colonial tuco-tuco is group living (burrow systems are routinely inhabited by multiple adults and their collective young) [17], the Patagonian tuco-tuco is solitary (burrow systems are never inhabited by more than one adult) [18]. Due to this pronounced difference in social behavior, these species have been the subjects of more than two decades of extensive field research regarding their behavior, ecology and demography.

**Fig 1:**
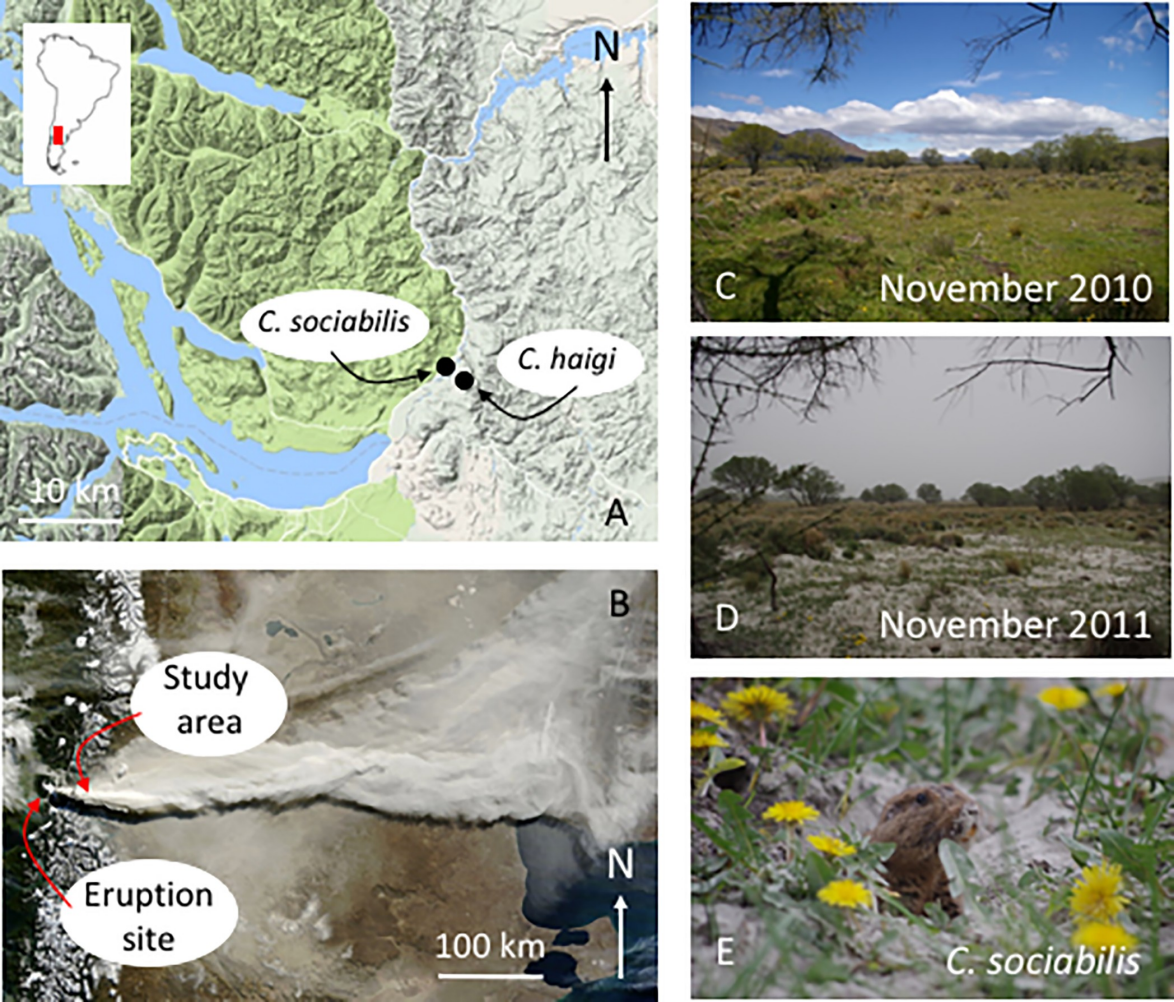
The study area in southwestern Argentina. In (A), the locations of the two study sites in the Limay Valley are indicated (inset: location of Limay Valley in Argentina). In (B), a photo of the ash plume produced by the Puyehue-Cordon Caulle volcano complex is shown, with the location of the study site relative to the volcano indicated (photo: NASA Earth Observing 1 satellite, 13 June 2011). (C) is a photo of the Rincon Grande study site taken in 2010. (D) is a photo taken from the same location in 2011, with the hills surrounding the study site obscured by falling ash. (E) is a photo of an adult female *C*. *sociabilis* foraging in the ash at the Rincon Grande study site in 2011.

The eruption of the Puyehue-Cordon Caulle volcano complex on 4 June 2011 had significant impacts on the Limay Valley and surrounding region. The initial eruption lasted 2 weeks, after which ash continued to fall intermittently until December 2011, resulting in a total release of more than 950 tons of volcanic debris [19,20]. The effects on the flora and fauna of the region were substantive, resulting in significant losses of livestock [21] and generating diverse negative outcomes for both native and exotic species [22–26]. The close proximity of the Limay Valley (~ 100 km east; Fig 1) to the site of the eruption meant that the populations of *C*. *sociabilis* and *C*. *haigi* that have been the focus of long-term study were also subject to extensive ash fall, providing a rare opportunity to assess directly the consequences of a severe environmental event on natural populations of native vertebrates. Analyses of different molecular markers have revealed contrasting information regarding post-eruption changes in genetic variability in these populations [27,28], highlighting the need for more detailed exploration of the effects of the eruption on the demography each study species. To identify the specific population parameters contributing to changes in genetic variation and, more generally, to characterize the immediate demographic consequences of the eruption on these animals, we compared pre- and post-eruption values for multiple elements of population structure including survival, offspring production, and juvenile recruitment. To relate these variables to potential environmental changes, we also examined pre- and post-eruption data on diet composition and availability of key food resources. Collectively, these analyses provide important insights into the specific factors contributing to observed post-eruption changes in population structure and genetic diversity in these species, with emphasis on the group-living *C*. *sociabilis*.

## Methods

### Study sites

The population of *C*. *sociabilis* studied occurred on Estancia Rincon Grande (40°58'30" S, 71°04'26" W), Neuquen Province, Argentina. The population of *C*. *haigi* studied was located on Estancia San Ramon (40°58'57" S, 71°04'12" W), Río Negro Province, Argentina (Fig 1). Despite occurring in different provinces, the two study sites were located directly across the Río Limay from one another, with a distance of < 1 km between sites. The Rincon Grande site consisted of an ~ 6 ha area of mesic grassland bounded to the east by the Río Limay and on all other sides by arid, steppe vegetation. The San Ramon site consisted of an ~ 4 ha area containing a mix of mesic and more arid steppe grassland bounded to the west by the Río Limay. This difference in the range of habitats encompassed by each site reflects ecological differences between the study species; while *C*. *sociabilis* is a habitat specialist that occurs primarily in mesic grasslands [15], *C*. *haigi* is more broadly distributed, occurring in both mesic and arid patches of habitat [14]. Comparisons of soil attributes, diet compositions, and the distributions of critical food resources revealed no significant differences between the Rincon Grande site and the mesic portion of the San Ramon site; the only significant habitat differences detected were between the wetter and drier portions of the San Ramon site [14].

### Demographic monitoring

Long-term monitoring of the study populations began in 1996 and consisted of an annual mark-recapture program combined with periodic surveys of vegetation and other habitat parameters [14,17,18,29]. Each year during the austral spring (November-December), we attempted to capture all members of each study population. While *C*. *sociabilis* were caught using hand-held nooses as described by [17] and [29], *C*. *haigi* were captured using plastic tube traps following the procedures of [18]. All capture localities were recorded using either a hand-held GPS unit (Garmin Etrex) or a georeferenced grid system (8 m x 8 m cell size) that had been established on each study site. Upon first capture, individuals were permanently marked by inserting a PIT tag (IMI-1000 Implantable Transponders, BioMedic Data Systems, Inc.) beneath the skin at the nape of the neck. Tags were read using a hand-held scanner (DAS 4004 Pocket Scanner, BioMedic Data Systems, Inc.), allowing us to follow known individuals across trapping efforts and field seasons. Captured individuals were weighed and their sex was determined; for females, apparent reproductive status (pregnant or lactating) was also recorded. Upon completion of these procedures, each animal was released at the point of capture. All procedures involving live animals were approved by the UC Berkeley Animal Care and Use Committee and conformed to the guidelines established by the American Society of Mammalogists for the use of wild mammals in research [30].

For both species, trapping at a given location typically continued until no further evidence of recent activity (e.g., freshly excavated soil, newly plugged burrow entrances) was detected [17,18]. Because *C*. *haigi* is solitary [18], determining when all individuals in a burrow system had been caught was straightforward; by comparing capture locations for adults to the locations of all burrow systems displaying evidence of recent activity, we estimated that each year > 95% of the animals resident on the San Ramon study site were captured. In contrast, because *C*. *sociabilis* is group living, determining when all residents of a burrow system had been caught was more challenging and often required temporarily (≤ 12 hours) holding animals in captivity [17]. Not all animals in some social groups could be captured; for these burrow systems, post-trapping observations of animals foraging were used to estimate the number, sexes, and relative ages (adult versus juvenile) of individuals that evaded capture [29]. Based on these observations and data on the number of captures per burrow system, we estimated that each year > 90% of the individuals in the study population at Rincon Grande were captured.

Pre-eruption, annual monitoring of the study populations continued until 2002 for *C*. *haigi* and until 2010 for *C*. *sociabilis*. Post-eruption monitoring was conducted during November-December 2011 (both species). Due to ongoing ash fall and the presence of extensive wind-blown ash in 2011, *C*. *haigi* were not captured in that year. Instead, post-eruption population density for this species was estimated based on the number of active (occupied) burrow systems on the study site. Active burrow systems were readily identified by the almost daily presence of fresh mounds of soil or fresh soil plugs at burrow entrances. Because *C*. *haigi* is solitary, with each burrow system occupied by only one adult [17], the number of active burrows provides a reliable estimate of the number of adults in the population. Although this procedure was not identical to the pre-eruption trapping protocol employed, the strict 1:1 correspondence between adults and burrow systems in this species suggests that post-eruption estimates of animal numbers were subject to minimal error. However, this method of estimating post-eruption population density–coupled with the gap in sampling for *C*. *haigi* after 2002 –limited our ability to compare pre- and post-eruption values for demographic parameters other than population density. Due to this constraint and due to our generally greater emphasis on the group-living *C*. *sociabilis* over the course of our pre-eruption monitoring, we emphasize analyses of this species in our comparisons of the pre- and post-eruption populations.

### Estimating demographic parameters

To assess the demographic consequences of the 2011 eruption, we compared pre- and post-eruption measures of annual population density (adults/ha) for each study species. To estimate population density, for each year of the study we divided the total number of adults (males and females) detected (captured and sighted) by the size of the area monitored during that year. All counts of adults were made during November-December and thus all estimates of population density corresponded to the same portion of the year.

Five additional demographic parameters–per capita female reproductive success, social group size, percent annual survival by yearling females, percent of adult females that were yearlings, and percent unmarked adult females in the focal population–were calculated for *C*. *sociabilis* only. We focused on data for females due to both the strongly female-biased sex ratio in *C*. *sociabilis* [29] and the ability to estimate female reproductive success directly by capturing juveniles at the time of weaning (i.e., genetic assignment of parentage not required). All females in a social group produce offspring, with each individual giving birth to a single litter of ~ 4 young per year during the austral spring, typically between October and December [31]. As a result, per capita female direct fitness was calculated by dividing the total number of juveniles captured in a burrow system by the total number of adult females resident in the same burrow system [31]. Although this procedure may have failed to capture within-group variation in offspring production, this limitation should not have influenced comparisons of pre- and post-eruption estimates of reproductive success.

Social group size was measured as the number of adult females per burrow system. Within years, group sizes typically ranged from one to six females [29], with this variation due primarily to among-group differences in rates of adult survival and juvenile recruitment. As a result, variation in mean annual group sizes reflects inter-year differences in these demographic parameters. Percent annual survival for yearling females was determined by comparing the identities of individuals captured in successive years. We focused on survival of yearling females to a second breeding season because yearlings typically comprise the majority (~ 66%) of adults in the study population [29] and thus sample sizes for this age cohort were large enough to provide robust estimates of pre- and post-eruption rates of survival. To maximize the accuracy of these estimates, only burrow systems trapped during two or more consecutive field seasons were included in analyses of female survival. Recruitment of juvenile females was assessed based on the percentage of yearling females in the population. Female *C*. *sociabilis* breed for the first time as yearlings [29] and thus their prevalence in the adult population reflects the recruitment of juveniles born during the previous year. Finally, the percentage of immigrant adult females in the population was determined based on captures of unmarked animals. Specifically, for burrow systems in which all individuals had been captured in a given year, capture of unmarked adult females at that location during the following year was interpreted as evidence of immigration [29].

### Statistical analyses

To assess the effects of the 2011 eruption on the demography of the focal study population of *C*. *sociabilis*, several statistical approaches were employed. Three of the demographic parameters examined (yearling female survival, proportion of yearlings in the population, proportion of unmarked females in the population) consisted of a single measure per year. For these variables, we examined differences between pre- and post-eruption years using Fisher's Exact tests. The remaining two demographic parameters (per capita female reproductive success, social group size) consisted of multiple data points per year. For these parameters, we constructed statistical models with the focal demographic trait as the response variable and “eruption period” (pre- or post-eruption) as a fixed effect. Data for per capita female reproductive success were normally distributed and thus we used a linear model for this analysis. In contrast, data for social group size were not normally distributed and thus we used a generalized linear model with a Poisson distribution to examine this variable. All statistical analyses were performed in R [32].

Because our data set included only one post-eruption year, the power of our linear models to detect differences in pre- and post-eruption values for individual demographic variables was limited. To address this and to examine the overall effects of the eruption on the structure of the study population, we performed an additional analysis in which we included all demographic parameters (yearling female survival, proportion of yearlings, proportion of unmarked females, per capita reproductive success, social group size) in a single model of demographic response, hereafter referred to as the “combined” model. Population density was also included in this analysis; because this parameter consisted of a single value per year, the significance of pre- versus post-eruption values could not be assessed using a separate model with density as the response variable. Prior to construction of the combined model, we converted the percentage of unmarked females per year to its converse–the percentage of marked females per year–so that all demographic variables were predicted to be negatively affected by the eruption. We then centered and standardized the yearly value for each demographic parameter and used the resulting data as the responses in a linear mixed effects model that simultaneously examined the effects of the eruption on each of these parameters. Eruption period (pre- or post-eruption) and year (included to account for annual variability in pre-eruption values) were used as fixed effects and the identity of each demographic parameter (e.g., group size, population density) was used as a random effect to control for potential differences in variability among the demographic variables examined. These analyses were conducted in the R package "lme4" [33].

### Vegetation surveys

To examine the impacts of the 2011 eruption on the food resources consumed by *C*. *sociabilis* and to explore possible links among ash fall, vegetation, and changes in specific demographic parameters, we compared pre- and post-eruption data regarding the prevalence of grasses in the genus *Poa*. Tuco-tucos are herbivorous and, based on microhistological analyses of fecal pellets, *Poa* comprises a substantive portion of the diets of both study populations [14]. For the focal population of *C*. *sociabili*s, the two other primary food resources consumed are grasses in the genus *Apera* and the herb *Acaena* (14, E. A. Lacey, pers. comm.). Data regarding the prevalence of *Poa* were collected at the Rincon Grande study site following the methods of [14]. Pre- and post-eruption data were collected during November-December 1999 and November 2011, respectively; despite the interval between these sampling periods, annual photo surveys suggested that vegetation on site remained relatively consistent until after the 2011 eruption. Measurements of *Poa* focused on the two most common vegetation types on the study site (described in results). For each of these vegetation types, the total number of blades of *Poa* was determined for multiple (N = 10 pre-eruption, 10 post-eruption) randomly selected 0.25 x 0.25 m quadrats; for each vegetation type, the same quadrats were used for both pre- and post-eruption counts and thus paired statistical tests were used to assess differences between time periods.

### Analyses of plant renewal

In addition to affecting the abundance of *Poa*, ash fall from the eruption may have impacted rates of renewal for this important food resource. Both study species forage by opening burrow entrances from underground, after which the animals crop the surrounding vegetation to a distance of about half a body length. Once all vegetation around a given entrance has been removed, the animal(s) in that burrow system open a new entrance at which to forage. As the grasses surrounding a used burrow entrance regrow, that location can again be used for foraging. As a result, the rate of renewal for food resources such as *Poa* is also an important component of the foraging ecology of the study animals.

To assess the effects of ash depth on rates of renewal for *Poa*, in November 2011 we established 6 experimental plots at the Rincon Grande study site. Each plot consisted of a 1.0 x 1.0 x 0.15 m cardboard enclosure, the interior of which was divided with cardboard partitions in to 16 equally-sized squares. All experimental plots were located at randomly selected locations within the two predominant vegetation types on the study site. Within each plot, 4 squares were randomly selected for use in this experiment (the remaining sections were used as part of a separate study). The ash in each selected square was carefully removed, after which the number of blades of *Poa* in each square was determined. To simulate foraging by tuco-tucos, all vegetation in the selected squares was then cut off at the level of the soil surface and removed from the plot. Each square was then filled with ash to a depth of 0, 5, 10 or 15 cm; the locations of the different ash depths were randomized across experimental plots.

After 2 weeks, the plots were revisited and the number of blades of *Poa* visible above the ash layer was determined for each square. Because the presence of ash may have affected the growth of *Poa*, these data capture information regarding renewal times for food resources rather than growth rates *per se*. To assess the effect of ash depth on renewal rates, we ran a linear mixed-effects model with the number of grass blades detected after 2 weeks as the dependent variable, ash depth and original (pre-cutting) number of grass blades as fixed effects, and plot identity as a random effect. The original number of grass blades was included to account for potential differences in *Poa* abundance among plots; plot identity was included to avoid potential pseudoreplication arising from unmeasured differences among plots. Analyses were conducted using the “lme4” package in R [33].

### Isotopic analyses of diet

To determine if potential changes in the vegetation available to the study animals impacted their diets, we compared stable isotope signatures for fur samples collected before and after the eruption. Due to the emphasis on Rincon Grande for vegetation analyses and the greater availability of pre-eruption samples for *C*. *sociabilis*, isotopic analyses focused on this study species. Pre-eruption samples were obtained by clipping small samples of fur from 6 specimens of *C*. *sociabilis* housed in the Museum of Vertebrate Zoology (MVZ 183315, 192236, 200348, 206878, EAL 178, EAL 179). All specimens were adult animals (accidental mortalities) that had been collected at Rincon Grande during October to December of 1994 through 2005. Post-eruption samples (N = 16) were obtained by clipping a similarly sized patch of fur from animals captured during November through December of the 2011 field season. In all cases, fur was removed from the rump, just anterior to the base of the tail, after which the animal was released at the point of capture The growth rate for fur in this species is ~ 0.5 cm per month (R. Takenaka, pers. comm.), suggesting that isotopic signatures from pre-eruption specimens were not confounded by seasonal differences in the timing since last molt and that isotopic signatures from post-eruption samples reflected food resources consumed after the June eruption. Prior to analysis, fur samples were washed using a 2:1 chloroform:methanol solution and then allowed to air dry for at least 24 hours [34]. Each cleaned sample (1.4–1.8 mg each) was packaged in a 5 x 9 mm tin capsule (Costech Analytical Tech, Inc.) for analysis. Samples from each individual were run in duplicate and the resulting values were averaged to obtain a mean value for each individual.

To relate isotopic signatures from fur samples to the food resources consumed by members of the Rincon Grande study population, we determined the δ^13^C and δ^15^N values for 2 of the primary food items (*Poa*, *Acaena*) consumed by the study animals, plus an additional 6 plant genera (*Carduus*, *Carex*, *Festuca*, *Senecio*, *Stipa*, *Taraxacum*) that each comprised a much smaller portion of the diet of the study population. Plant samples (N = 11 for *Acaena*, N = 20 for *Poa*, N = 10 for all other genera) were collected during December 2011; due to the absence of *Apera* on the study site during this year, this genus was not included in our isotopic analyses. Samples were air dried in the field and stored in paper envelopes until they could be analyzed. Prior to analysis, each sample was oven dried to remove any remaining moisture and then homogenized using a bead beater. Between 4.0 and 6.7 mg of each sample was then packaged as described above for fur samples.

For both fur and plant samples, δ^13^C and δ^15^N values were determined by continuous flow isotope ratio mass spectrophotometry using a CHNOS Elemental Analyzer (vario ISOTOPE cube, Elementar, Hanau, Germany) and IsoPrime 100 mass spectrophotometer (Isoprime Ltd, Cheadle, UK). Analyses were performed in the Center for Stable Isotope Biogeochemistry (CSIB) at the University of California, Berkeley. Stable isotope abundances are presented in δ notation as deviations from standard references. References used were atmospheric nitrogen (AIR) and Vienna PeeDee Belemnite (V-PDB) for δ^15^N and δ^13^C values, respectively. Abundances are given in parts per thousand (‰) according to the following equation: δX = (R_sample_/R_standard_)–1, where X represents ^15^N or ^13^C and R is the ratio of the heavy to the light isotope (e.g., ^15^N/^14^N). The external precision for C and N isotope determinations (1 sigma standard deviation) was ± 0.10‰ and ± 0.15‰, respectively.

Isotopic signatures for pre- and post-eruption fur samples were compared using Welch’s T-tests [35] as implemented in R, with separate analyses performed for δ^13^C and δ^15^N values. To estimate the relative contribution of each plant genus to the overall diet of *C*. *sociabilis*, we used the Bayesian mixing model SIAR [36]. Because taxon-specific trophic enrichment factors were not available for *Ctenomys*, we used the experimentally validated, published trophic enrichment values for *Mus musculus*; the values used were 1.10‰ for carbon [37] and 3.05‰ for nitrogen [38]. We conducted separate SIAR analyses for pre- and post-eruption samples, after which we compared the SIAR outputs for each plant taxon using Welch’s T-tests as implemented in R. Mixing models produce more accurate diet estimates when dietary sources vary widely in isotopic signatures (e.g., as in an omnivorous diet) and when experimentally derived, taxon-, tissue-, and diet-specific trophic enrichment factors are used [39]. Given these limitations, we focused our analyses on relative changes in diet composition before versus after the 2011 eruption.

## Results

Both study sites experienced substantial ash fall, with ash blanketing the entire Limay region during the 2011 field season (Fig 1). Measurements of ash depth at 10 randomly selected locations per study site revealed that mean depth at Rincon Grande was 4.2 ± 0.7 cm versus 3.4 ± 0.6 cm at San Ramon. This difference was significant (Mann-Whitney U test, Z = 2.645; N = 10 per site, two-tailed p = 0.008: S1 Table), indicating that deposition of ash at Rincon Grande was greater than at San Ramon. This finding is consistent with the relative locations of these study sites with respect to the Puyehue-Cordon Caulle complex that was the source of the ash (Fig 1).

### Demographic changes

**Fig 2:**
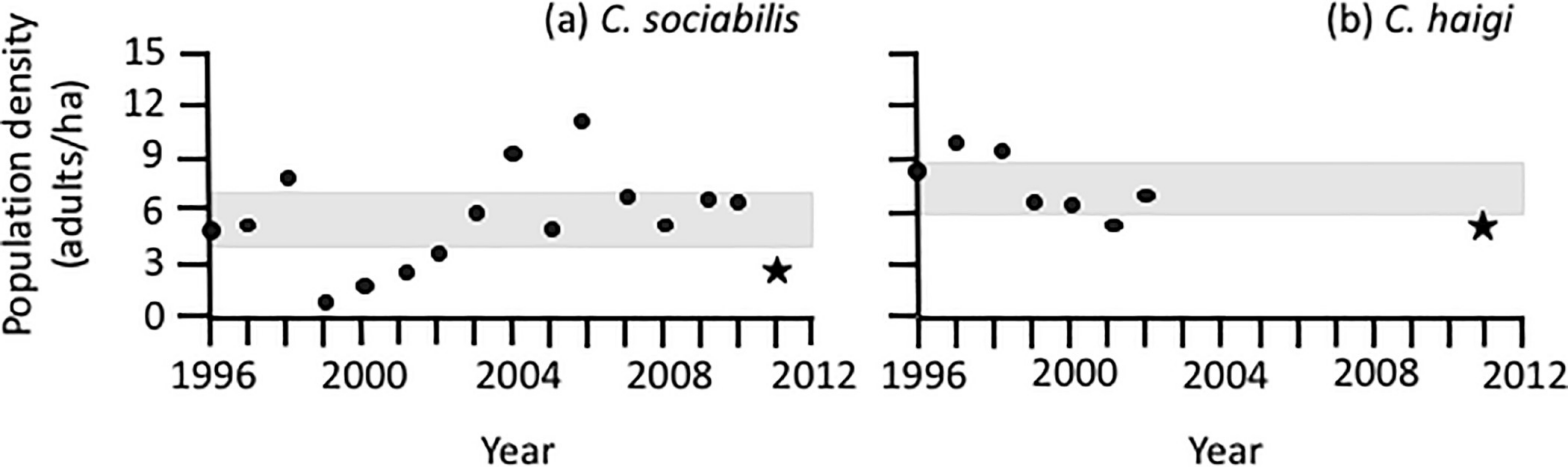
**Comparisons of pre- and post-eruption measures of population density for (A) *C*. *sociabilis* and (B) *C*. *haigi*.** For each year of the study, density was calculated as the number of adults in the study population divided by the area (ha) monitored. Pre-eruption measures of density (N = 15 and 7 years) are indicated with circles; the single post-eruption measure for each population (from 2011) is indicated with a star. The 95% confidence interval for pre-eruption measures of density for each population is indicated by the gray rectangle.

The densities of both study populations were reduced after the 2011 eruption (Fig 2). The post-eruption density for *C*. *sociabilis* (3.2 adults/ha) represented a 40.1% decrease relative to the mean for pre-eruption years (5.3 ± 2.9 adults/ha, N = 15 years; S2 Table). Interestingly, population density in 2011 was not the lowest recorded during this study (Fig 2); lower densities were recorded during 1999–2001, indicating that this population had experienced other pronounced reductions in the recent past. The post-eruption density for *C*. *haigi* (5.5 adults/ha) was 25.4% lower than the mean pre-eruption density for this population (12.1 ± 2.8, N = 7 years). In contrast to *C*. *sociabilis*, the post-eruption density for *C*. *haigi* was tied for the lowest recorded for this species during the course of this study (S2 Table). Although the presence of only a single post-eruption value per species precluded statistical analyses of these contrasts, 2011 densities for both species fell outside the 95% confidence intervals for pre-eruption data, suggesting that post-eruption decreases in population densities were significant.

**Fig 3:**
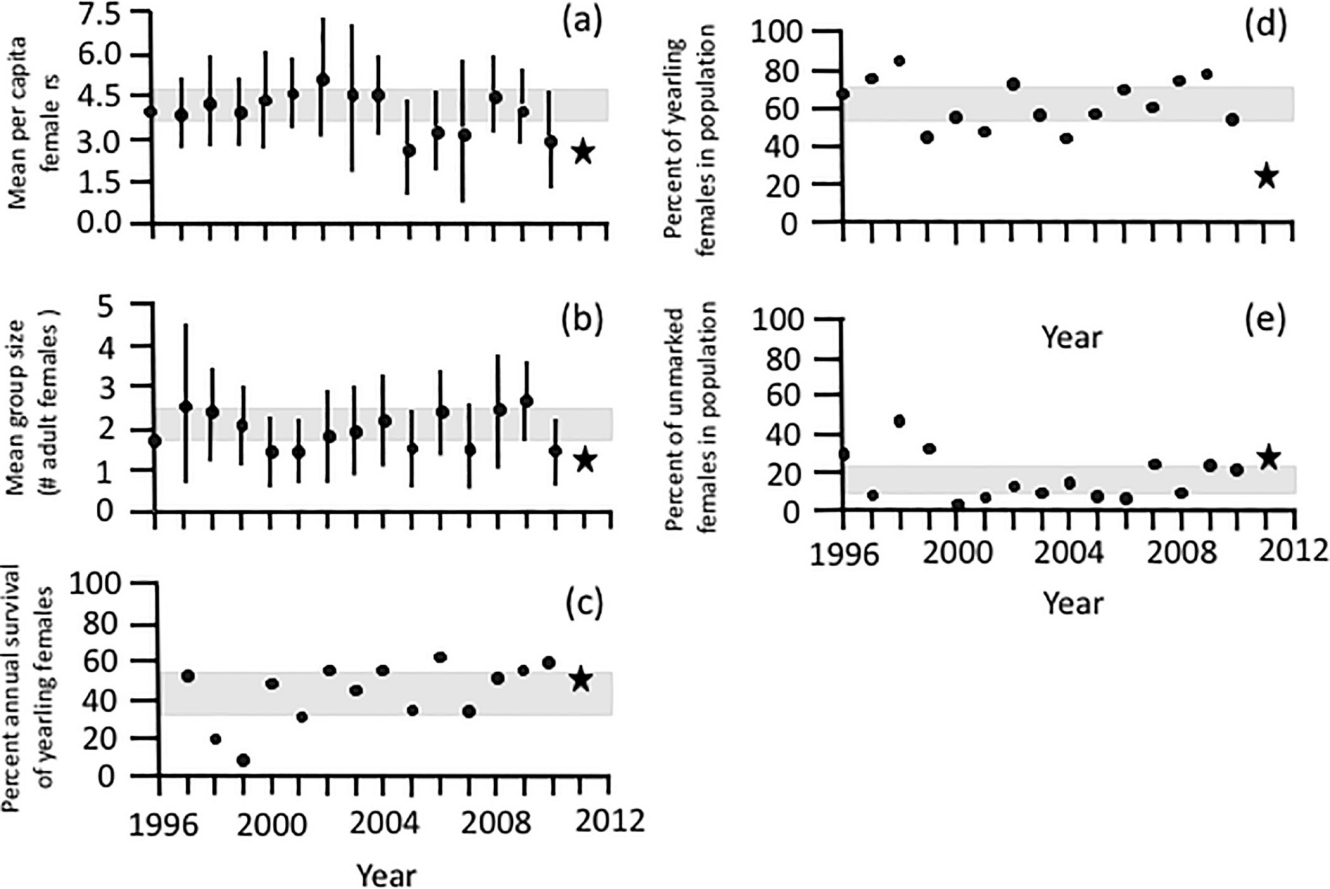
Pre- and post-eruption measures of social structure and demography in *C*. *sociabilis*. Mean (± 1 SD) annual values are shown for two parameters: (A) per capita female reproductive success and (B) social group size. Annual values for (C) percent survival of yearling females, (D) percentage of yearlings in the breeding population of females, and (E) percentage of unmarked females in the population are also shown. Pre-eruption measures (N = 15 years) are indicated with circles; the single post-eruption measure for each parameter (from 2011) is indicated with a star. The 95% confidence interval for pre-eruption measures of each parameter is indicated with a gray rectangle.

In *C*. *sociabilis*, no significant differences were found between pre- and post-eruption values for social group size, annual survival of yearling females, or the percentage of unmarked females in the study population (Table 1; Fig 3; S3 and S4 Tables). In contrast, the per capita number of pups reared to weaning in 2011 (2.7 ± 0.6, N = 5 social groups) was significantly less than that in pre-eruption years (3.9 ± 1.3, N = 100 social groups over 15 years: Table 1; Fig 3; S4 Table). Similarly, the proportion of yearling females in the population in 2011 (25.0% of 12 known-age females) was significantly lower than in previous years (65.2 ± 15.5%, N = 297 known-age females over 15 years: Table 1, Fig 3; S3 Table).

**Table 1:** Analyses of demographic parameters measured before and after the 2011 volcanic eruption.

Variable	Test	Effect ± SE	test statistic	*P*
Yearling female survival	Fisher's exact	NA	NA	0.725
Prop. unmarked females in the pop.	Fisher's exact	NA	NA	0.572
Prop. yearlings in the pop.	Fisher's exact	NA	NA	**0.001**
Per capita reproductive success	LM	1.58±0.59	t = 2.66	**0.009**
Group size	GLM	0.33±0.38	z = 0.86	0.389
Combined: Eruption (pre vs. post ^1^)	LMM	1.04±0.49	t = 2.15	**0.035**
Combined: Year	LMM	-0.001±0.026	t = -0.02	0.983

^1^ Comparison of pre-eruption to post-eruption values; the positive effect indicates that values were higher in the pre- versus the post-eruption period.

The combined model of demographic responses included yearling female survival, proportion of marked females in the population, proportion of yearling females in the population, per capita reproductive success, social group size, and population density, with all variables centered and standardized (S5 and S6 Tables). LM = linear model; GLM = general linear model; LMM = linear mixed effects model. *P* values that are significant at α = 0.05 are indicated in bold.

In the linear mixed model that included data for all demographic parameters, eruption period had a significant effect on demography, with pre-eruption values for these parameters being larger than post-eruption values (Table 1). In contrast, year of data collection did not significantly affect any of the demographic parameters considered (Table 1). Thus, both our parameter-specific and combined analyses of demographic variables were consistent in identifying significant changes between pre- and post-eruption years of the study.

### Impacts on vegetation

In 2011, visual inspection of the Rincon Grande study site indicated that *Poa* was present, having grown up through the layer of ash that covered the site (Fig 1). In contrast, several other typically abundant plants were absent from the site, presumably because they were unable to grow through the ash layer. Notably, *Apera* was not detected on the study site in 2011; *Acaena* was present but was largely covered by ash. The first vegetation type examined consisted of a mix of *Poa*, dandelion (*Taraxacum*), and reeds (*Juncus*). Prior to the eruption, the mean number of blades of *Poa* in this vegetation type was 77.7 (± 41.3) per 0.25 m^2^ while the post-eruption value was 173.6 (± 94.5) blades per 0.25 m^2^; this difference was significant (paired t-test, T = 2.5, N = 10,10, two-tailed p = 0.034; S7 Table). The second vegetation type examined consisted of a mix of *Poa*, *Erodium* and, for a portion of the spring, *Apera*. Mean pre- and post-eruption counts of *Poa* for this vegetation type consisted of 14.0 (± 15.0) blades and 55.3 (± 72.0) blades per 0.25 m^2^, respectively; this difference was not significant (Wilcoxon Signed Rank Test, Z = -1.26, N = 10,10, two-tailed p = 0.208; S7 Table). Thus, while the abundance of *Poa* was generally greater after the eruption, this difference was only significant for the vegetation type consisting of a mix of *Poa*, *Taraxacum*, and *Juncus*.

### Effects of ash depth on food resource renewal

**Fig 4:**
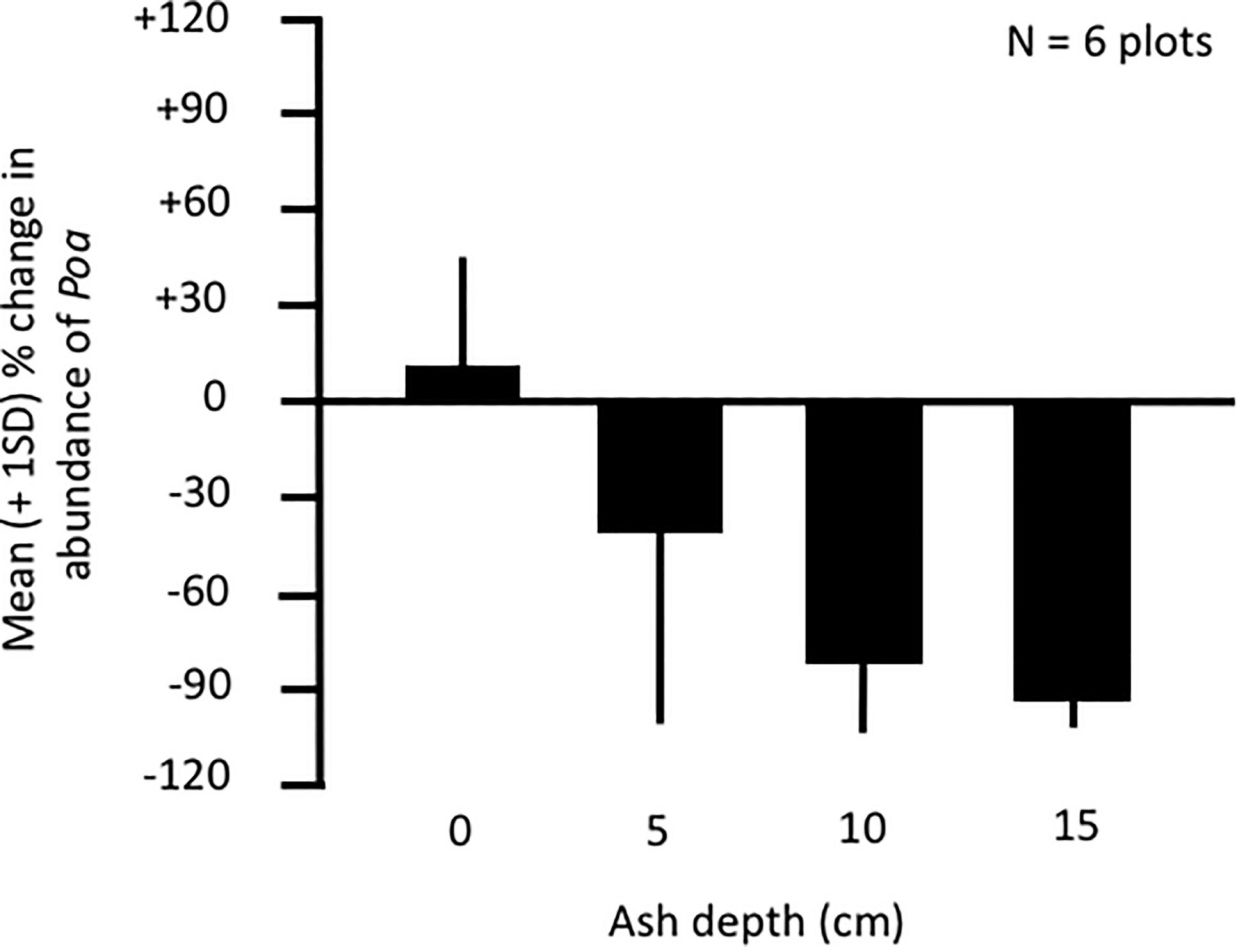
Impacts of ash depth on the regrowth of grasses in the genus *Poa*. Data are from 6 experimental plots established at the Rincon Grande study site in December 2011. Each plot consisted of a series of subplots that ranged in ash depth from 0 to 15 cm. Two weeks after plots were established, the number of blades of *Poa* visible above the ash surface was counted for each subplot; these values are expressed as the mean (± 1 standard deviation) percent change in the number of blades present before versus after the manipulation of ash depth.

Data from our experimental plots revealed that above ground re-emergence of *Poa* was positively associated with the original (pre-cutting) number of blades in a plot (effect ± SE = 0.214 ± 0.070, t = 3.05, p = 0.006) but was negatively associated with ash depth (effect ± SE = -2.390 ± 0.395, t = -6.04, p < 0.001; Fig 4; S8 Table).

### Stable isotope comparisons of diets

**Fig 5:**
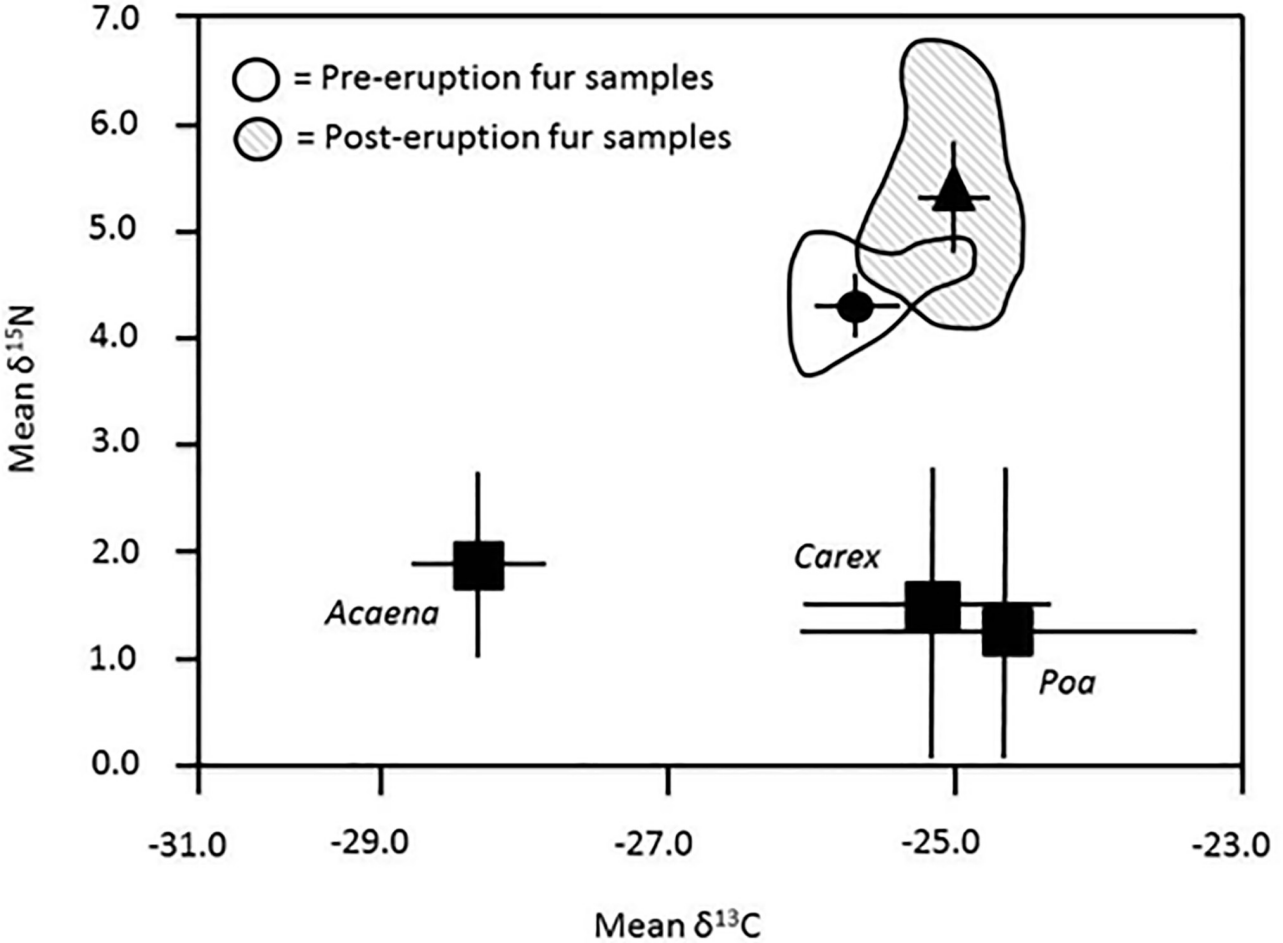
Comparisons of pre- and post-eruption stable isotope signatures for fur samples collected from *C*. *sociabilis*. For both sampling periods, mean (± 1 SD) values for δ^13^ C and δ^15^ N are shown; the distribution of individual data points from pre- and post-eruption samples are indicated by the irregular shapes surrounding these means. For comparison, mean (± 1 SD) for δ^13^ C and δ^15^ N values for three genera of plants comprising important components of the diet of the study population are also shown. Values are shown for pre-eruption fur samples (circles), post-eruption fur samples (triangles), and plant samples (squares).

Stable isotope analyses of fur samples revealed that both δ^13^C and δ^15^N differed significantly between pre- and post-eruption samples (δ^13^C: Welch’s t-test, t = -3.3601, N = 6, 16, df = 6.79, two-tailed p = 0.0126; δ^15^N: Welch’s t-test, t = -3.9848, N = 6,16, df = 14.24, two-tailed p = 0.0013; Fig 3, S9 Table). Although pre-eruption samples (N = 6) had been collected over a period of 2 decades, variances in both δ^13^C and δ^15^N values for these samples were comparable to or less than the variances for post-eruption samples (N = 16) collected during a single field season (Fig 5), suggesting that our comparisons were not confounded by the extended time period encompassed by the pre-eruption data set.

**Fig 6:**
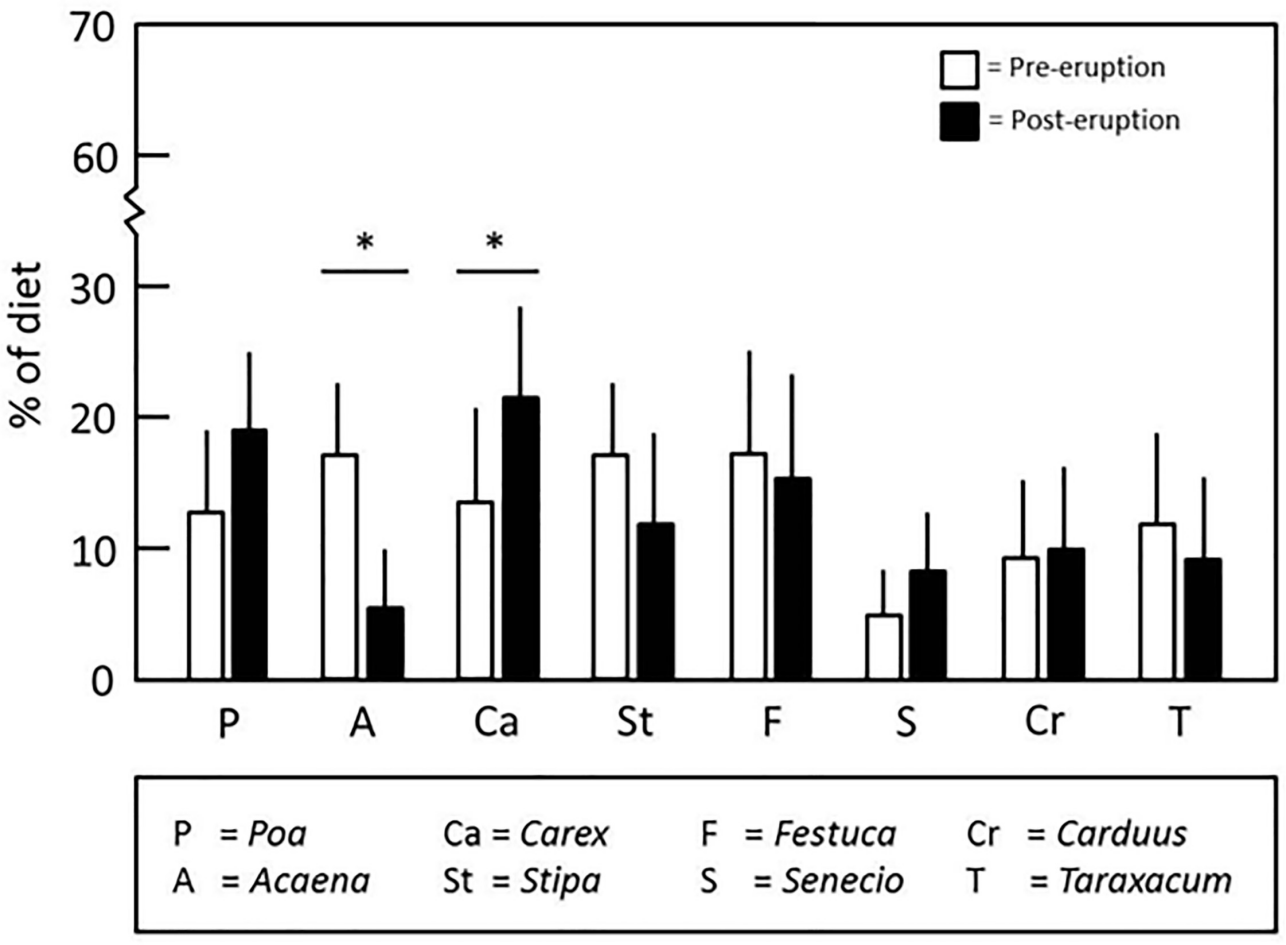
Comparisons of pre- and post-eruption diets of the study population of *C*. *sociabilis*. Results of SIAR analyses of δ^13^ C and δ^15^ N signatures from fur samples. Data for 8 genera of plants consumed by the study population are shown; significant pre- and post-eruption contrasts are indicated with an asterisk (Welch’s T-tests, both p < 0.044). SIAR analyses were conducted using trophic enrichment factors obtained from *Mus musculus* (see methods).

Data from analyses of fur samples were consistent with observed post-eruption changes in the vegetation at the study site for *C*. *sociabilis*. Output from our SIAR analyses (Fig 6) revealed that while *Poa* consumption increased after the eruption, this difference was not significant (Welch’s t-test, t = 1.8041, N = 6, 16, df = 8, two-tailed p = 0.1089; S10 Table). In contrast, the percentage of the diet composed of *Acaena* decreased significantly after the eruption (Welch’s t-test, t = 3.4131, N = 6, 16, df = 6, two-tailed p = 0.0143; S10 Table). Comparisons of the third major pre-eruption food resource (*Apera*) were not conducted due to the absence of this genus in 2011, which precluded collection of samples for the SIAR analyses. Of the 6 other plant genera examined, only *Carex* displayed a significant change in prevalence in the diet of the study animals, with this genus being significantly more abundant in the post-eruption diet of *C*. *sociabilis* (Welch’s t-test, t = 2.3355, N = 6, 16, df = 9, two-tailed p = 0.0443; S10 Table). These dietary changes, in particular the reduced consumption of *Acaena*, are consistent with the shift to less negative δ^13^C values for fur samples collected after the 2011 eruption (Fig 6).

## Discussion

Our analyses revealed that the 2011 eruption of the Puyehue-Cordon Caulle volcano complex had immediate significant demographic impacts on *Ctenomys* in the Limay Valley of northern Patagonia. Notably, post-eruption population densities for *C*. *sociabilis* and *C*. *haigi* were markedly reduced compared to densities for pre-eruption years, with this decline being greater for *C*. *sociabilis*. The more extensive data set for *C*. *sociabilis* revealed a significant post-eruption decrease in per capita female reproductive success and a significant post-eruption decrease in the percentage of yearling females in the study population for this species. These demographic differences were associated with changes in diet composition that paralleled pre- and post-eruption differences in the availability of two key food resources, namely *Poa* and *Apera*. In contrast, other demographic parameters such as social group size and survival of adult females did not appear to be affected by the eruption. Because our data set was limited to a single post-eruption year, we were not able to assess the longer term consequences of this event on the demography or ecology of our study animals and thus the more enduring, potential evolutionary implications of the eruption remain unknown.

### Demographic impacts

The greater percent decline in population density detected for *C*. *sociabilis* suggests that the consequences of the 2011 eruption were more pronounced for this species, an interpretation that is consistent with the typically greater depth of ash on the study site for *C*. *sociabilis*. The lower proportion of yearling females in the post-eruption population of *C*. *sociabilis* suggests that reduced recruitment of juvenile females from 2011 to 2012 contributed to the observed decrease in population density. Although the lack of data from *C*. *haigi* for the years immediately preceding the eruption precluded direct interspecific comparisons of rates of juvenile recruitment during this period, we note that the 2011 eruption, which began in early June, coincided with the approximate end of the annual period of juvenile dispersal for *C*. *sociabilis* (E. A. Lacey, pers. comm.). In contrast, dispersal by juvenile *C*. *haigi* tends to occur earlier during the austral autumn, with individuals establishing residence in new burrow systems by April or May (E. A. Lacey, pers. comm.). While dispersal in both species remains poorly characterized, the timing of the eruption leads us to suspect that this event had greater impacts on juvenile recruitment and, accordingly, population density in *C*. *sociabilis*.

*C*. *sociabilis* was also characterized by a significant post-eruption reduction in per capita female reproductive success. Multiple factors may have contributed to this decrease, particularly given that prior to eruption, mean per capita female reproductive success appeared to decline from 2008 to 2010. While we cannot exclude the potential effects of other, non-eruption related factors on variation in per capita female fitness, we note that the relatively limited number of offspring produced in 2011 coincided with apparent post-eruption changes in available food resources. Stable isotope analyses revealed differences in pre- and post-eruption diets that were consistent with observed changes in vegetation, in particular the reduced post-eruption availability of *Acaena* and *Apera*; although the relative abundance of *Poa* was greater after the eruption, the delayed post-eruption renewal of this critical food item may also have affected the resources available to the study animals. These changes may, in turn, have affected production of offspring during the first breeding season following the eruption due to reductions in food availability, food quality, or both. The interval between the first release of ash in June and our field sampling in November-December 2011 encompassed much of the annual reproductive cycle of *C*. *sociabilis*, including most of the period during which adult females in the study population were lactating [31,29]. For females, lactation is typically the energetically most costly portion of the mammalian reproductive cycle [40] and thus it seems plausible that reduced availability of key food resources during this period would have impacted reproductive success. Breeding females captured during the 2011 field season appeared to be in poor condition, with extensive patches of missing pelage (E. A. Lacey and M. N. Tammone, pers. comm.), thereby lending potential anecdotal support to the hypothesis that changes in food resources impacted female reproductive success during the first breeding season following the eruption.

### Implications for genetic variation

Previous studies [27, 28] have revealed that the 2011 eruption affected levels of genetic variability in both study species and a primary objective of our analyses was to identify the demographic parameters that may have contributed to these changes in genetic variation. Loss of genetic variability following anthropogenically-induced bottlenecks has been documented for a number of mammalian species, including elephant seals [41], fur seals [42], black-footed ferrets [43], arctic foxes [44], lions [45], and Florida panthers [46]. In comparison, the genetic impacts of natural catastrophic events appear to have received relatively little attention, due at least in part to limited opportunities to evaluate the immediate effects of such phenomena. Exceptions include studies of two species of tuco-tucos (*C*. *maulinus* and *C*. *coyhaiquensis*) from southern Chile, populations of which experienced significant reductions in size due to volcanic eruptions occurring within the last few decades. Allozyme analyses of tissue samples collected before and after these events indicate that the eruption of Volcan Lonquimay in 1988 was associated with a significant reduction in allozyme variation in 3 populations of *C*. *maulinus* [47]. Similarly, the eruption of Volcan Hudson in 1991 resulted in a significant reduction in allozyme variation in *C*. *coyhaiquensis* [48]. Although pre- versus post-eruption population sizes were known for *C*. *maulinus* [47,48], no other demographic data were available for these species and thus it was not possible to relate the observed changes in genetic variability to eruption-induced changes in specific demographic parameters.

In the present study, we found pronounced declines in population density for both *C*. *sociabilis* and *C*. *haigi* during the first breeding season following the 2011 eruption. These reductions may have resulted in the stochastic elimination of alleles from each study population [49]. More specifically, a post-eruption decrease in density–in particular the number of reproductive females–should have resulted in a reduction in the effective size (N_e_) of each study population [49,50]. In *C*. *sociabilis*, the effects of this decrease may have been exacerbated by the post-eruption reduction in the number of yearling females breeding for the first time. Collectively, these findings lead us to expect that post-eruption genetic variability in each population should also have been reduced. Analyses of pre- and post-eruption samples from the study species using a genome-wide panel of single nucleotide polymorphisms (SNPs) support this prediction, revealing small but significant decreases in genetic variability in both study populations [28]. These findings indicate that abrupt reductions in population size of the magnitude reported here (25–50%) can produce significant changes in genetic variability over short time scales. Volcanic eruptions, including eruptions of the Puyehue-Cordon Caulle complex, have occurred repeatedly in northwestern Patagonia [51,52], suggesting that *C*. *sociabilis* and *C*. *haigi* have likely undergone multiple such fluctuations in demography and, potentially, genetic structure over the course of their evolutionary histories.

### Conclusions

The 2011 eruption of the Puyehue-Cordon Caulle volcano complex had multiple significant effects on the study populations, including impacts on ecology, demography, and genetic variability. Based on the relative declines in population density, the effects of the eruption appeared to be greater for the group-living *C*. *sociabilis*. The more detailed demographic data set available for this species revealed that this decline in population density was due primarily to a reduction in the recruitment of juvenile females from 2010 to 2011. As a result, most reproduction during 2011 was by older females that had bred during the previous year. Post-eruption changes in population density and–at least for *C*. *sociabilis*–the age structure of breeding females are expected to have altered effective population size and thus contributed to observed reductions in genetic variability in the study populations. Collectively, these findings suggest that even in the absence of local extinctions, significant environmental events can produce demographic changes that impact genetic variability and, thus, potential patterns of future evolutionary change.

## Supporting information

S1 Table Depth of ash fall on the study sites for *C*. *sociabilis* (Rincon Grande) and *C*. *haigi* (San Ramon). For each site, ash depth (in cm) was recorded for 10 randomly selected locations.
(PDF)

S2 Table Annual measures of population density (adults/hectare) for the study populations of *C*. *sociabilis* and *C*. *haigi*. Density was determined by dividing the number of adults detected by the area in which trapping occurred.
(PDF)

S3 Table Annual measures of demographic parameters for *C*. *sociabilis*. Parameters examined were the percentage of yearling females in the population, the percentage of yearling females that survived from the previous to the current breeding season, and the percentage of unmarked adult females in the population. For percent yearling survival, the sample size (# of yearlings in previous season) is given in parentheses. Between years, sample sizes for yearling survival and the age composition of the population differed depending on which social groups were captured in their entirety in successive years.
(PDF)

S4 Table Annual measures of social group size (number of adult females) and per capita female reproductive success (number of pups reared to weaning) for *C*. *sociabilis*. (PDF)

S5 Table Centered and standardized values for the demographic variables used in the combined linear modeling analysis. (PDF)

S6 Table Correlations among the centered and standardized demographic variables used in the combined linear modeling analysis. (PDF)

S7 Table Pre- and post-eruption abundance of *Poa* on the study site for *C*. *sociabilis*. Counts represent the number of blades of *Poa* per 0.25 x 0.25 m^2^ quadrat for two predominant vegetation types on the study site (described in detail in the primary text). The same sampling quadrats were used before and after the eruption.
(PDF)

S8 Table Renewal of *Poa* in relation to ash depth. Data are from 6 experimental plots, each containing four 0.25 x 0.25 m^2^ subplots. In each plot, the number of *Poa* blades was counted (T = 0), after which all blades were cut off at the soil surface and subplots were filled with ash to a depth of 0, 5, 10, or 15 cm. After two weeks (T = 2), the number of blades of *Poa* visible above the ash surface was counted and this number used to calculate percent change in available *Poa*.
(PDF)

S9 Table Stable isotope signatures for pre- and post-eruption fur samples from *C*. *sociabilis*. Values shown are means for duplicate (N = 2) samples analyzed per individual.
(PDF)

S10 Table Stable isotope signatures for plant genera used in SIAR analyses of pre- and post-eruption diets for *C*. *sociabilis*. (PDF)
