# Supplementary material for: Ecological and demographic impacts of a recent volcanic eruption on two endemic patagonian rodents
Source: PLoS One. 2019 Mar 7;14(3):e0213311. doi: 10.1371/journal.pone.0213311 (PMC6405110; doi:10.1371/journal.pone.0213311)
Supplement: S1 Table — For each site, ash depth (in cm) was recorded for 10 randomly selected locations. (PDF) [file pone.0213311.s001.pdf]

S1 Table.

	Depth of ash (cm)	
	Rincon Grande	San Ramon
Sample #		
1	4.0	4.0
2	2.7	3.5
3	4.0	2.7
4	4.3	2.9
5	4.5	3.2
6	5.0	2.4
7	4.2	4.0
8	4.3	3.0
9	3.7	3.9
10	5.0	3.9
