# Supplementary material for: Ecological and demographic impacts of a recent volcanic eruption on two endemic patagonian rodents
Source: PLoS One. 2019 Mar 7;14(3):e0213311. doi: 10.1371/journal.pone.0213311 (PMC6405110; doi:10.1371/journal.pone.0213311)
Supplement: S2 Table — Density was determined by dividing the number of adults detected by the area in which trapping occurred. (PDF) [file pone.0213311.s002.pdf]

S2 Table

	Density (adults/hectare)	
Year	*C. sociabilis*	*C. haigi*
1996	4.9	8.7
1997	5.1	10.3
1998	8.1	9.4
1999	0.8	7.4
2000	1.1	6.5
2001	2.1	5.5
2002	3.2	6.8
2003	6.0	*
2004	9.1	*
2005	4.9	*
2006	11.3	*
2007	7.0	*
2008	5.1	*
2009	6.8	*
2010	6.5	*
2011	3.2	5.5
Area monitored:	~ 5.3 ha	~ 3.2 ha

* No data available for this year
