# Supplementary material for: Ecological and demographic impacts of a recent volcanic eruption on two endemic patagonian rodents
Source: PLoS One. 2019 Mar 7;14(3):e0213311. doi: 10.1371/journal.pone.0213311 (PMC6405110; doi:10.1371/journal.pone.0213311)
Supplement: S3 Table — Parameters examined were the percentage of yearling females in the population, the percentage of yearling females that survived from the previous to the current breeding season, and the percentage of unmarked adult females in the population. For percent yearling survival, the sample size (# of yearlings in previous season) is given in parentheses. Between years, sample sizes for yearling survival and the age composition of the population differed depending on which social groups were captured in their entirety in successive years. (PDF) [file pone.0213311.s003.pdf]

**S3 Table**.

| Year | % yearling female survival | # known age females | % yearling females in population | % unmarked females in population |
|---|---|---|---|---|
| 1996 | * | 9 | 66.7 | 30.8 |
| 1997 | 53.8 (13) | 29 | 79.3 | 6.5 |
| 1998 | 20.0 (10) | 7 | 85.7 | 46.2 |
| 1999 | 7.1 (28) | 23 | 47.8 | 32.4 |
| 2000 | 50.0 (4) | 18 | 55.6 | 0.0 |
| 2001 | 33.3 (6) | 24 | 50.0 | 4.0 |
| 2002 | 57.1 (14) | 34 | 70.6 | 8.1 |
| 2003 | 44.8 (29) | 33 | 57.6 | 5.7 |
| 2004 | 56.3 (16) | 23 | 43.5 | 14.8 |
| 2005 | 36.0 (25) | 21 | 57.1 | 4.5 |
| 2006 | 61.9 (21) | 30 | 70.0 | 3.2 |
| 2007 | 35.0 (20) | 18 | 61.1 | 25.0 |
| 2008 | 52.9 (17) | 13 | 76.9 | 7.1 |
| 2009 | 53.8 (13) | 6 | 100.0 | 45.5 |
| 2010 | 57.0 (7) | 9 | 33.3 | 35.7 |
| 2011 | 50.0 (8) | 12 | 25.0 | 29.4 |

* No data available for this year
