# Supplementary material for: Ecological and demographic impacts of a recent volcanic eruption on two endemic patagonian rodents
Source: PLoS One. 2019 Mar 7;14(3):e0213311. doi: 10.1371/journal.pone.0213311 (PMC6405110; doi:10.1371/journal.pone.0213311)
Supplement: S4 Table — (PDF) [file pone.0213311.s004.pdf]

S4 Table.

Year	Social group size (# females)	Per capita female reproductive success
1996	1	3.0
	6	2.3
	1	4.0
	1	5.0
	1	4.0
	1	4.0
1997	4	3.3
	4	2.5
	3	3.6
	1	5.0
	3	3.6
1998	1	5.0
	1	5.0
	1	5.0
	1	3.0
	5	3.0
	3	3.3
	4	2.0
	1	6.0
	5	3.6
	2	3.6
1999	1	4.0
	1	2.0
	1	4.0
	3	4.0
	3	3.0
	2	4.0
2000	1	3.0
	3	1.6
	3	4.3
	2	5.0
	1	6.0
	1	5.0
	2	4.5
	1	3.0

Year	Social group size (# females)	Per capita female reproductive success
2003	2	4.0
	1	3.0
	2	2.5
	1	6.0
	1	1.0
	1	6.0
	3	3.6
	4	4.3
	3	3.3
	1	6.0
	1	6.0
2004	1	5.0
	1	5.0
	3	5.0
	1	5.0
	6	3.2
	2	4.5
	3	4.0
	1	6.0
	2	3.0
2005	1	1.0
	2	2.5
2006	2	3.0
	2	5.0
	5	2.8
	1	2.0
	1	3.0
	5	3.2
	1	4.0
	3	2.0
	1	4.0
	2	4.5
	1	4.0
	4	2.7
	3	3.0
	3	2.6

Year	Social group size (# females)	Per capita female repro-ductive success
2001	2	5.0
	1	5.0
	3	3.3
	1	4.0
	1	5.0
	1	5.0
	2	4.0
	1	6.0
	1	3.0
	1	4.0
2002	4	3.0
	1	4.0
	1	6.0
	1	3.0
	1	5.0
	3	3.6
	4	2.3
	2	6.0
	1	3.0
	1	6.0
	1	7.0
	5	4.0
	1	5.0
	1	6.0
	1	6.0
	1	6.0
2007	1	5.0
	3	3.0
2008	1	3.0
	4	3.7
	1	4.0
	1	5.0
	1	4.0
	1	5.0
2009	3	3.0
	1	4.0
2010	3	2.7
	1	4.0
	1	1.0
	2	0.5
2011	1	2.0
	1	3.0
	2	3.0
	2	2.5
	1	1.3
