# Supplementary material for: Ecological and demographic impacts of a recent volcanic eruption on two endemic patagonian rodents
Source: PLoS One. 2019 Mar 7;14(3):e0213311. doi: 10.1371/journal.pone.0213311 (PMC6405110; doi:10.1371/journal.pone.0213311)
Supplement: S5 Table — (PDF) [file pone.0213311.s005.pdf]

S5 Table.

Year	Eruption period	Group size	Reproductive success	% Marked females	% Yearlings	Yearling survival
1996	Pre	-0.559599	0.1258272	-0.7574806	0.28031684	NA
1997	Pre	1.38944898	-0.008312	0.76138718	0.92987861	0.59492609
1998	Pre	1.1718593	0.39295922	-1.7200553	1.25981475	-1.5907806
1999	Pre	0.35937019	-0.122961	-0.8574884	-0.6940258	-2.4249705
2000	Pre	-0.8234843	0.50760815	1.16766869	-0.2919162	0.34919575
2001	Pre	-0.9785749	0.9432741	0.9176493	-0.5806103	-0.7307244
2002	Pre	-0.2633067	1.30327175	0.66137942	0.48137167	0.80832349
2003	Pre	-0.098957	0.62798953	0.81139106	-0.1888111	0.01293318
2004	Pre	0.21816837	1.04875112	0.24259694	-0.9157017	0.75659078
2005	Pre	-0.9253348	-2.1293173	0.88639687	-0.2145874	-0.5561266
2006	Pre	0.50983113	-0.385507	0.96765318	0.45044016	1.11871971
2007	Pre	-1.1429245	0.45028369	-0.3949525	-0.0083773	-0.6207924
2008	Pre	1.25982109	0.58442294	0.72388427	0.80615256	0.5367268
2009	Pre	1.83851704	-0.122961	-1.6763019	1.99701581	0.59492609
2010	Pre	-0.6683938	-1.7853705	-1.0637544	-1.4415374	0.8018569
2011	Post	-1.2864411	-1.4299588	-0.6699738	-1.8694233	0.34919575
