# Supplementary material for: Ecological and demographic impacts of a recent volcanic eruption on two endemic patagonian rodents
Source: PLoS One. 2019 Mar 7;14(3):e0213311. doi: 10.1371/journal.pone.0213311 (PMC6405110; doi:10.1371/journal.pone.0213311)
Supplement: S6 Table — (PDF) [file pone.0213311.s006.pdf]

S6 Table.

Demographic variable	Group size	Reproductive success	% marked females	Yearling survival
Rep. success	-0.088			
% marked	0.345	-0.34		
Yearling survival	-0.191	0.167	-0.414	
% yearlings	-0.748	-0.198	-0.107	0.05
