# Supplementary material for: Ecological and demographic impacts of a recent volcanic eruption on two endemic patagonian rodents
Source: PLoS One. 2019 Mar 7;14(3):e0213311. doi: 10.1371/journal.pone.0213311 (PMC6405110; doi:10.1371/journal.pone.0213311)
Supplement: S7 Table — Counts represent the number of blades of Poa per 0.25 x 0.25 m2 quadrat for two predominant vegetation types on the study site (described in detail in the primary text). The same sampling quadrats were used before and after the eruption. (PDF) [file pone.0213311.s007.pdf]

S7 Table.

	Vegetation type 1			Vegetation type 2	
	Pre-eruption	Post-eruption		Pre-eruption	Post-eruption
Sample			Sample		
1	27	93	1	15	0
2	98	164	2	5	6
3	106	78	3	25	52
4	122	114	4	1	137
5	112	90	5	0	0
6	96	123	6	20	0
7	0	320	7	4	121
8	108	231	8	0	0
9	61	337	9	40	38
10	47	186	10	35	199
