# Supplementary material for: Ecological and demographic impacts of a recent volcanic eruption on two endemic patagonian rodents
Source: PLoS One. 2019 Mar 7;14(3):e0213311. doi: 10.1371/journal.pone.0213311 (PMC6405110; doi:10.1371/journal.pone.0213311)
Supplement: S8 Table — Data are from 6 experimental plots, each containing four 0.25 x 0.25 m2 subplots. In each plot, the number of Poa blades was counted (T = 0), after which all blades were cut off at the soil surface and subplots were filled with ash to a depth of 0, 5, 10, or 15 cm. After two weeks (T = 2), the number of blades of Poa visible above the ash surface was counted and this number used to calculate percent change in available Poa. (PDF) [file pone.0213311.s008.pdf]

S8 Table.

Sample	Ash depth (cm)	# of *Poa* blades T = 0 weeks	= 2 week	% change
1	0	60	45	-25.0
	5	130	28	-78.5
	10	104	40	-61.5
	15	93	10	-89.2
2	0	50	57	14
	5	42	5	-88.1
	10	80	10	-87.5
	15	64	9	-95.3
3	0	17	27	58.8
	5	21	34	61.9
	10	15	6	-60.0
	15	46	3	-93.5
4	0	13	17	30.8
	5	18	9	-50.0
	10	7	0	-100.0
	15	18	0	-100.0
5	0	30	45	21.6
	5	14	7	-50.00
	10	33	1	-97.00
	15	37	3	-91.9
6	0	69	40	-42.00
	5	58	16	-72.4
	10	40	2	-95.0
	15	73	0	-100.0
