# Supplementary material for: Ecological and demographic impacts of a recent volcanic eruption on two endemic patagonian rodents
Source: PLoS One. 2019 Mar 7;14(3):e0213311. doi: 10.1371/journal.pone.0213311 (PMC6405110; doi:10.1371/journal.pone.0213311)
Supplement: S9 Table — Values shown are means for duplicate (N = 2) samples analyzed per individual. (PDF) [file pone.0213311.s009.pdf]

S9 Table.

	$\delta^{13}C$	δ15N	MVZ specimen number	Collection date
Pre-erupt	-25.59	4.50	183315	6-Dec-94
	-25.06	4.86	192236	7-Nov-99
	-25.53	4.58	200348	15-Nov-02
	-25.89	4.88	206878	25-Oct-03
	-25.6	4.25	EAL 178	19-Nov-05
	-25.91	3.96	EAL 179	5-Dec-05

	$\delta^{13}C$	δ15N	Animal ID	Collection date
Post-erup	-24.84	5.17	3572	6-Nov-11
	-25.33	4.76	6804	8-Nov-11
	-25.25	4.68	7E 01	11-Nov-11
	-25.09	4.86	026A	11-Nov-11
	-24.98	4.42	25 24	12-Nov-11
	-25.29	6.44	3F28	14-Nov-11
	-25.21	5.36	1E 38	14-Nov-11
	-24.94	6.10	2361	14-Nov-11
	-25.19	5.51	7D4D	14-Nov-11
	-25.36	5.41	4C70	16-Nov-11
	-25.26	5.74	7A3E	16-Nov-11
	-25.15	5.26	3168	16-Nov-11
	-25.55	4.81	204D	25-Dec-11
	-25.05	5.43	5D5D	27-Dec-11
	-24.79	4.98	O218	29-Dec-11
	-24.92	5.84	O649	30-Dec-11
