# Supplementary material for: Ecological and demographic impacts of a recent volcanic eruption on two endemic patagonian rodents
Source: PLoS One. 2019 Mar 7;14(3):e0213311. doi: 10.1371/journal.pone.0213311 (PMC6405110; doi:10.1371/journal.pone.0213311)
Supplement: S10 Table — (PDF) [file pone.0213311.s010.pdf]

S10 Table.

Plant genus	$\delta^{13}C$	δ15N	Plant genus	$\delta^{13}C$	δ15N	Plant genus	$\delta^{13}C$	δ15N
Acaena	2.23	-28.08	*Carex*	-24.43	-0.37	*Senecio*	-29.66	7.84
	2.39	-28.91		-27.32	2.76		-30.37	4.98
	2.36	-28.33		-24.67	2.34		-29.9	5.08
	0.71	-28.04		-25.08	2.28		-30.5	5.98
	2.36	-28.38		-25.58	-0.47		-29.22	4.37
	1.71	-28.73		-25.38	1.55		-29.04	6.15
	1.39	-27.77		-26.32	1.72		-30.65	5.28
	-0.11	-28.65		-25.5	3.09		-28.56	4.21
	3.13	-27.79		-25.38	0.45		-29.45	4.47
	2.1	-28.23		-23.53	2.20		-29.65	5.98
	1.99	-28.71						
			Festuca	-25.93	2.17	*Stipa*	-25.57	1.22
Carduus	-25.09	2.16		-26.74	-1.28		-28.35	0.83
	-29.16	3.22		-26.72	0.83		-26.32	0.08
	-26.62	1.72		-26.52	2.46		-23.96	0.01
	-26.13	0.27		-25.84	1.66		-24.29	0.15
	-28.61	5.14		-25.99	0.63		-26.71	0.43
	-28.51	3.97		-25.59	1.50		-24.79	0.46
	-27.99	2.83		-26.23	-0.58		-25.93	1.96
	-27.99	4.18		-26.23	0.60		-26.12	-1.22
	-29.49	1.82		-24.81	2.78		-25.51	2.62
	-28.75	2.63						

Plant genus	$\delta^{13}C$	δ15N	Plant genus	$\delta^{13}C$	δ15N
Poa	-25.94	0.34	*Taraxacum*	-26.35	2.89
	-26.42	-1.21		-27.68	3.53
	-25.15	2.22		-28.06	1.39
	-25.69	0.79		-28.37	3.17
	-25.48	0.29		-27.42	1.86
	-24.73	2.15		-28.65	3.06
	-25.65	1.86		-27.89	5.16
	-27.05	4.06		-28.07	2.90
	-24.63	1.14		-27.54	-1.67
	-26.44	1.09		-27.54	0.30
	-23.23	3.50			
	-23.34	1.37			
	-21.83	3.43			
	-23.23	0.33			
	-24.93	0.43			
	-23.55	0.86			
	-24.01	0.47			
	-23.04	-0.70			
	-26.26	2.16			
	-23.35	1.29			
